# Comparison of Tissue Architectural Changes between Radiofrequency Ablation and Cryospray Ablation in Barrett's Esophagus Using Endoscopic Three-Dimensional Optical Coherence Tomography

**DOI:** 10.1155/2012/684832

**Published:** 2012-07-10

**Authors:** Tsung-Han Tsai, Chao Zhou, Hsiang-Chieh Lee, Yuankai K. Tao, Osman O. Ahsen, Marisa Figueiredo, Desmond C. Adler, Joseph M. Schmitt, Qin Huang, James G. Fujimoto, Hiroshi Mashimo

**Affiliations:** ^1^Department of Electrical Engineering and Computer Science, and Research Laboratory of Electronics, Massachusetts Institute of Technology, Cambridge, MA 02139, USA; ^2^Gastroenterology Section, VA Boston Healthcare System, Boston, MA 02130, USA; ^3^Harvard Medical School, Boston, MA 02115, USA; ^4^LightLab Imaging Inc.-St. Jude Medical Inc., Westford, MA 01886, USA

## Abstract

Two main nonsurgical endoscopic approaches for ablating dysplastic and early cancer lesions in the esophagus have gained popularity, namely, radiofrequency ablation (RFA) and cryospray ablation (CSA). We report a uniquely suited endoscopic and near-microscopic imaging modality, three-dimensional (3D) optical coherence tomography (OCT), to assess and compare the esophagus immediately after RFA and CSA. The maximum depths of architectural changes were measured and compared between the two treatment groups. RFA was observed to induce 230~260 *μ*m depth of architectural changes after each set of ablations over a particular region, while CSA was observed to induce edema-like spongiform changes to ~640 *μ*m depth within the ablated field. The ability to obtain micron-scale depth-resolved images of tissue structural changes following different ablation therapies makes 3D-OCT an ideal tool to assess treatment efficacy. Such information could be potentially used to provide real-time feedback for treatment dosing and to identify regions that need further retreatment.

## 1. Introduction

Radiofrequency ablation (RFA) and cryospray ablation (CSA) are recently developed methods that utilize thermal gradients to treat dysplastic and early cancer lesions of the esophagus, such as those arising in the setting of Barrett's esophagus (BE). Both therapeutic technologies allow broad and superficial treatment fields for BE [1–8]. Recent clinical trials using RFA treatment have shown that complete eradication of dysplasia (CE-D) was achieved in 98% and 93% of patients with low-grade dysplasia (LGD) and high-grade dysplasia (HGD) at two-year followup [9]. Complete eradication of intestinal metaplasia (CE-IM) was achieved in 92% of patients with nondysplastic BE (NDBE) at up to 5 years of followup [10]. CSA is a newer therapeutic technology, so fewer large-scale clinical trials have been conducted. However, several pilot studies have shown that CSA is highly effective in eradicating HGD [6–8]. Complete eradication of the HGD, CE-D, and CE-IM in 94%, 88%, and 53% of BE patients was reported in a multicenter study [8]. Another multicenter study also reported 97%, 87%, and 57% complete eradication of HGD, CE-D, and CE-IM at 10.5 months of followup [7].

Although these studies indicate that both therapeutic technologies allow broad and superficial treatment fields for BE, repeated RFA/CSA treatments were generally required to achieve complete treatment response. On average, CE-IM was achieved after over 3.4 sessions using RFA [4, 9, 10] and 4.2 sessions using CSA [8, 11] for patients with BE. Considering the recovery time between consecutive therapeutic procedures (6–8 weeks for RFA treatment and 4–6 weeks for CSA treatment), the overall treatment process to achieve CE-IM can be over a year long [7–10, 12, 13]. Multiple repeated esophagogastroduodenoscopy (EGD) and therapeutic procedures also increase costs to the healthcare system [14–16]. Thus, methods to improve the efficacy of each therapeutic procedure would reduce the total number of treatment sessions, reduce patient anxiety, and improve the benefit from these endoscopic therapeutic techniques.

Optical coherence tomography (OCT) is a volumetric imaging technique that generates cross-sectional images of internal structure with micrometer resolutions and millimeter imaging depth by measuring the echo time delays of backscattered light [17]. Endoscopic OCT techniques have been developed to image the human gastrointestinal (GI) tract with over 1 mm imaging depth [18–27] and were extensively used in esophagus to evaluate specialized intestinal metaplasia, dysplasia, and adenocarcinoma with high sensitivity and specificity to differentiate different pathologies [23, 28]. Recently, with the dramatic increases of imaging speed, endoscopic 3D-OCT has become possible and provides a powerful combination of high-resolution, large field of view and rapid data acquisition [24–27] and has been used to image patients with upper and lower GI diseases [24, 27, 29]. The objective of this investigation is to assess differences in architectural changes in esophageal tissues following RFA and CSA, using real-time endoscopic 3D-OCT so that such evaluation may help improve the treatment efficacy in the future.

## 2. Materials and Methods

### 2.1. Patient Enrollment and Study Protocol

This study was conducted at the Veterans Affairs Boston Healthcare System (VABHS), Jamaica Plain Campus, and the study protocol was approved by the VABHS, Harvard Medical School, and the Massachusetts Institute of Technology. Patients in this study were recruited from volunteers aged from 30 to 80 undergoing diagnostic endoscopy and biopsy for Barrett's esophagus at VABHS based on a history of a biopsy positive for moderate-grade dysplasia within the previous 18-month period. Patients were diagnosed with standard white light endoscopy, and the length of visible BE was recorded based on the Prague C&M criteria [30]. Informed consent was obtained from each patient. Patients were assigned to receive RFA or CSA treatments based on physician judgment. For each RFA treatment, the patient received two sets of ablations using the BARRX Halo90 catheter. A treatment set consisted of 300 Watts at 12 J/cm^2^ applied twice for any treatment surface and repeated consecutively to adjacent areas until the entire BE region was ablated. Desquamated epithelium was removed by rigorous scraping between the two sets of ablations, per standard protocol set by the manufacture. 3D-OCT imaging was performed before and immediately after each set of ablations. For each CSA treatment, the CSA Medical system was used. The tissue area was sprayed with liquid nitrogen to freeze for 20 seconds, allowed to thaw completely, and then followed by another 20 seconds freezing. 3D-OCT imaging was performed before and immediately after the completion of CSA.

### 2.2. Endoscopic 3D-OCT Imaging

A prototype endoscopic 3D-OCT system developed in collaboration with LightLab Imaging-St. Jude Medical (Westford, MA, USA) was used for this study [26, 31]. The system had a lateral resolution of 15 *μ*m, an axial resolution of 5 *μ*m, and imaging depth of ~2 mm in tissue. 3D-OCT imaging was performed with the OCT catheter introduced through the biopsy channel of the endoscope (GIF Q180; Olympus, Tokyo, Japan), enabling simultaneous video endoscopy. Volumetric OCT data was acquired at 60,000 axial lines per second and 60 frames per second. The imaging catheter scanned a helical pull-back pattern with an 8 mm circumference and 20 mm pull-back length within 20 seconds. The imaging catheter was placed at 6 o'clock in the endoscopic field, and the pull-back imaging overlapped the GEJ. For RFA patients, multiple 3D-OCT datasets were acquired at the GEJ before the ablation treatments, immediately after the first set of RFA ablations (but prior to vigorous scraping of the desquamated epithelium), and after the second set of RFA ablations. For CSA patients, multiple 3D-OCT data sets were similarly obtained before the ablation treatments and immediately after the entire CSA treatment for each endoscopic session.

### 2.3. Image Analysis

Each 3D-OCT data set was reviewed and analyzed after the endoscopy session using a 3D rendering software (Amira, Visage Imaging, Inc.). The volumetric data was cylindrically shaped but was converted to rectangular form by unfolding [26] to better visualize *en face* features. For each patient, tissue morphological changes at 3~5 locations from the 3D-OCT data sets were identified as regions of interest (ROIs). The maximum depth of architectural changes was measured for each ROI, and comparison was made between the two treatment groups.

## 3. Results

**Figure 1:**
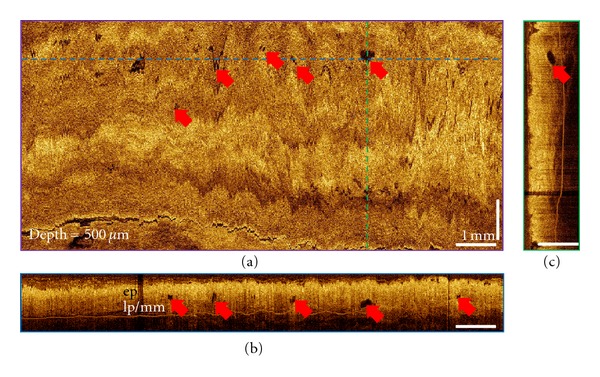
3D-OCT orthoplanes from the BE region before RFA treatment. (a) The *en face* orthoplane is located at a depth of 500 *μ*m. The colored dotted lines indicate location of complementary orthoplanes (b) and (c). Red arrows indicate the locations of the BE glands. (b) The cross-sectional YZ orthoplane shows the epithelium (ep), lamina propria (lp), and muscularis mucosa (mm). (c) The cross-sectional XZ orthoplane shows the hyposcattering feature of the BE glands on top of the lp/mm layer.

**Figure 2:**
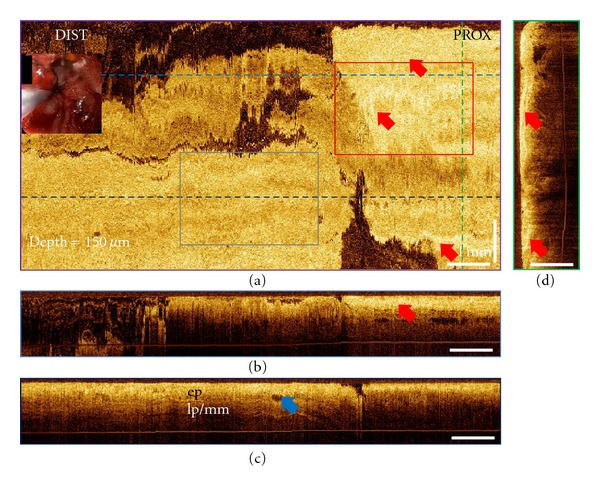
3D-OCT orthoplanes from the BE region immediately after RFA treatment. (a) The *en face* orthoplane is located at a depth of 150 *μ*m. The colored dotted lines indicate location of complementary orthoplanes (b), (c), and (d). Red arrows indicate the locations of the burned tissue on top of the treated regions, and the blue arrow indicates the location of the residual gland. (b-c) The cross-sectional YZ orthoplanes show the epithelium (ep), lamina propria (lp), and muscularis mucosa (mm). (d) The cross-sectional XZ orthoplane shows the burned tissue right on top of the muscularis mucosal layer. Inset shows a video endoscopy image with the 3D-OCT probe in position prior to image acquisition. Red box and gray box indicate regions of interest in Figures 3(a)–3(c) and Figures 3(d)–3(f), respectively.

3D-OCT imaging was performed on patients following RFA (*n* = 10) and CSA (*n* = 3) treatments for BE with the BARRX Halo90 system and the CSA Medical system, respectively. There were no adverse events after the RFA or CSA treatments. Representative 3D-OCT volumetric datasets obtained from the treated site of a 70-year-old patient before and right after RFA treatment are shown in Figures 1 and 2. The patient was diagnosed with 3 cm BE with HGD around the gastroesophageal junction (GEJ) based on the biopsy taken 4 months before the treatment. These three orthoplanes show 3D-OCT data spanning the RFA treatment site immediately after the second set of ablations, with colored dotted lines indicating the locations of the complementary planes. At a depth of 150 *μ*m, the *en face* orthoplane shows clear delineation between regions with and without hyperscattering features, which are consistent with burned esophageal tissues. The burned tissues were recognized by conventional endoscopic examination as patches of white debris.

**Figure 3:**
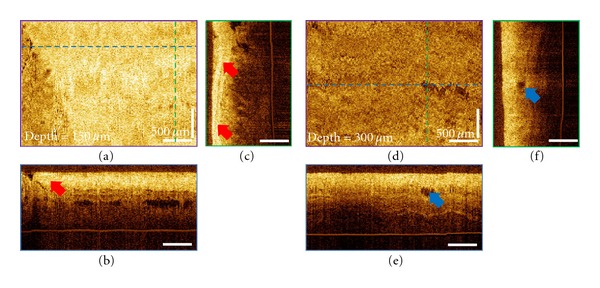
Magnified orthoplanes of the treated area showing the hyperscattering feature of the burned tissue as the red arrows indicated and the hyposcattering feature of the residual BE glands as the blue arrows indicated.

As reported in a previous study, cross-sectional orthoplanes revealed clear differences in layered architecture in the esophagus [24]. In this study, the cross-sectional imaging capability was also used to characterize the tissue morphologic changes after the ablation therapies.  Figure 3 shows the magnified orthoplanes of the ROI marked in Figure 2. Superficial burned tissues showed hyperscattering features. The endoscopic imaging field was covered with blood and tissue debris immediately after the RFA treatment, so it is difficult to evaluate the presence of residual glands or unburned BE using white light endoscopy or NBI due to the limited visibility. Areas with incomplete ablation can be distinguished in the OCT images taken immediately after the RFA. Figures 3(d)–3(f) show the magnified orthoplanes of the ROI (gray box) marked in Figure 2. Hyposcattering features of typical BE glands can be observed at the edge of the ablated area, indicating this location might require further treatment. At the followup two months later, the BE length was reduced to 1 cm around GEJ, and most of the prior treated site was covered with neosquamous tissue.

Among all the patients receiving RFA therapy, the depth of architectural changes was measured to be 263.3 *μ*m (SD = 31.4 *μ*m) after the first set of the ablation and 237 *μ*m (SD = 34.9 *μ*m). A complete cross-sectional fly-through is shown in Video 1 (see Video 1 in Supplementary material available online at doi:10.1155/2012/684832). 

**Figure 4:**
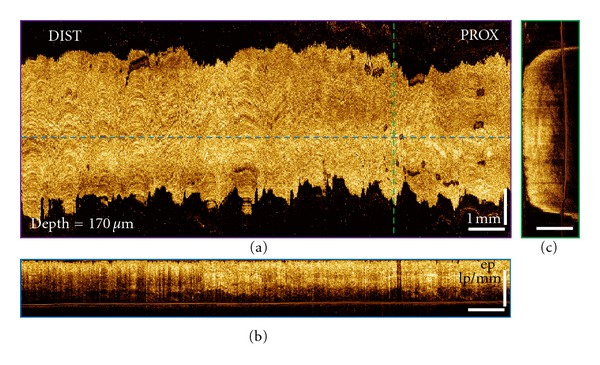
3D-OCT orthoplanes from the BE region before CSA treatment. (a) The *en face* orthoplane is located at a depth of 170 *μ*m. The colored dotted lines indicate location of complementary orthoplanes (b) and (c). (b) The cross-sectional YZ orthoplane shows the epithelium (ep), lamina propria (lp), and muscularis mucosa (mm). (c) The cross-sectional XZ orthoplane shows the typical BE structure.

**Figure 5:**
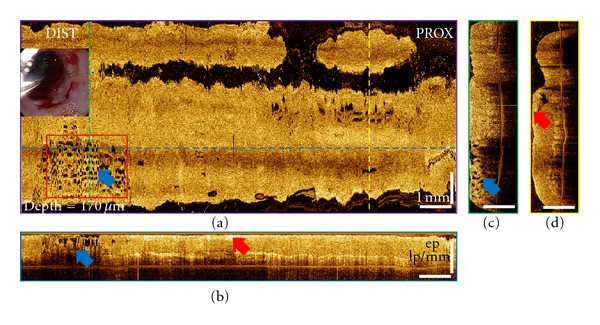
3D-OCT orthoplanes from the BE region immediately after CSA treatment. (a) The *en face* orthoplane is located at a depth of 170 *μ*m. The colored dotted lines indicate location of complementary orthoplanes (b), (c), and (d). Blue arrows indicate the locations of the edema-like tissues, and red arrows indicate the hyperscattering necrotic tissues on top of the treated regions. (b) The cross-sectional YZ orthoplane shows the epithelium (ep), lamina propria (lp), and muscularis mucosa (mm). (c) The cross-sectional XZ orthoplane shows the edema-like tissue extending into the muscularis mucosal layer. (d) The cross-sectional XZ orthoplane shows the hyperscattering, detached tissue on top of the treated area. Inset shows a video endoscopy image with the 3D-OCT probe in position prior to image acquisition. Red box indicates region of interest in Figure 6.

**Figure 6:**
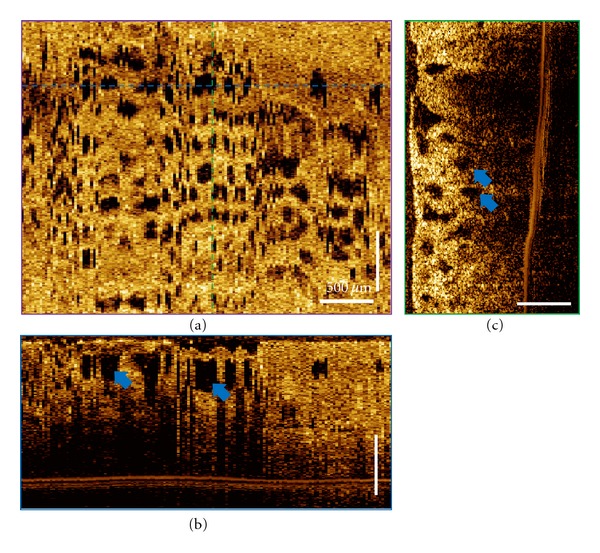
Magnified orthoplanes of the treated area showing the spongiform appearance due to the edematous tissue caused by freezing, as indicated by blue arrows.

Representative of 3D-OCT volumetric datasets of the treated site from a 72-year-old patient before and immediately after CSA treatment are shown in Figures 4 and 5. The patient was diagnosed with 4 cm BE with HGD in the distal esophagus based on the biopsy taken 9 months before the treatment. These three orthoplanes show 3D-OCT data spanning the CSA treatment site immediately after the procedure. In some ablated regions, thin hyperscattering structures can be observed on top of the tissue. At a depth of 170 *μ*m, regions with edema-like changes can be clearly observed from the *en face* OCT images in some areas of the ablation field. Conventional endoscopy shows the distal esophagus after the tissue was thawed completely.  Figure 6 shows the magnified orthoplanes of the ROI marked with a red box in Figure 5. In the OCT images taken immediately after the CSA treatment, there was no structural feature observed that indicates areas that require reablation. However, at the followup 3 months later, there was still BE distributed at the distal 1/3 esophagus with neosquamous tissue covering ~60% of the previously treated sites. 

Compared to RFA, there was less desquamation, but edema-like changes creating a “spongy” appearance observed as hyposcattering structures reaching a depth of 650 *μ*m in certain areas of the ablation field. Complete cross-sectional fly-through is shown in Video 2. These results suggest that 3D-OCT imaging can be a useful tool for providing immediate feedback about therapy-induced architectural changes on esophageal tissues.

The comparison between RFA and CSA is summarized in Table 1. In all patients treated with RFA, burned and desquamated tissues appeared hyperscattering, measuring 263.3 *μ*m (SD = 31.4 *μ*m) in thickness. However, the depth of structural changes measured after the second RFA set may not reflect the actual depth of the second RFA ablation if tissue was incompletely removed after the first RFA. The second RFA set at the same site caused structural changes with depth of 237 *μ*m (SD = 34.9 *μ*m). In patients of the CSA group, tissue architectural changes with edema-like “spongy” appearance caused by freezing were observed measuring 643 *μ*m (SD = 30 *μ*m) in depth. Compared to CSA, RFA caused more visible architectural changes observed on 3D-OCT imaging (including sloughing mucosa and more visible debris). However, the spongiform architectural changes caused by CSA appear deeper.

## 4. Discussion

With the advent of endoscopic ablative therapies for dysplastic and early cancer lesions in the gastrointestinal tract, methods to visualize the treatment field and optimize therapy will be in demand. While endoscopic 3D-OCT does not possess the same magnification or contrast as conventional histopathologic analysis, it is capable of visualizing tissue microstructures *in vivo *over a large field of view and provides real-time tissue depth information at micron-scale resolution. RFA was observed to induce about 230~260 *μ*m depth of architectural changes for each application, while CSA was observed to induce edema-like spongiform changes to 660 *μ*m deep. The depth of tissue structural changes for both therapies meets the required depth of tissue destruction for BE [32], which was confirmed by histology from 100 cases of BE patients. The ability to detect micron-scale tissue structural changes during the ablation therapy sessions makes 3D-OCT an ideal tool to assess treatment efficacy and may provide real-time feedback for treatment dosing and for identifying regions that need additional treatments. The correlation of the depth of such physical structural changes on OCT to actual depth of biological cell death and the extent of persistent tissue destruction compared to transient thermal effects remain unclear and will require histological correlates from samples taken during the ablation session. However, we did observe consistently the growth of neosquamous epithelium over the treated sites upon endoscopic followup visits after both RFA and CSA treatments, suggestive of a persistent and eventually similar tissue architectural change after both treatment modalities. In order to further study the acute treatment response at the cellular level, future studies will require correlation of architectural changes identified by OCT immediately after the ablation with histological analysis of deep jumbo forceps biopsies from the same locations and perhaps use of biomarkers of necrosis and apoptosis.

Traditional assessment of the efficacy of the ablation therapies requires from weeks to months for patients to revisit the clinics [12, 13, 33]. Patients would require retreatment if residual BE is observed endoscopically, including the regions that were missed in the previous treatment session [4, 8–11]. Endoscopic 3D-OCT may enhance the real-time assessment of BE patients prior to and following ablative therapies such as RFA and CSA by enabling comprehensive imaging of tissue microstructure over a large surface area. The increased analysis volume and high imaging acquisition speed of 3D-OCT may be used to guide ablation, with improved imaging of subsurface tissue structure in real time. To date, there is no other real-time imaging technology that can achieve the high imaging speed, broad imaging coverage, and subsurface visibility while having the spatial resolution that can show detail structural information from the tissue. Moreover, OCT is not dependent on contrast agents, as in confocal endomicroscopy, which would seep and obliterate the view during ablative therapies. Using endoscopic 3D-OCT, the datasets can be acquired in 20 seconds and reviewed immediately, suggesting the possibility of performing volumetric assessment of treated sites immediately after the treatment, improvement of the treatment efficacy with the guided ablation, and better quality of life with the reduction of the number of treatment sessions.

## 5. Conclusion

Endoscopic 3D-OCT can identify structural changes after RFA and CSA treatments, including the burned, hyperscattering tissues and the spongiform regions. These characteristics may be used to assess the treatment efficacy of ablation therapies. 3D-OCT may provide valuable information which will enable endoscopists to make real-time treatment decisions to improve the efficacy of RFA and CSA treatment and reduce the number of required treatments in the future.

## Supplementary Material

Supplementary Video 1: Complete cross-sectional fly-through of the volumetric OCT images obtained immediately after the RFA treatment. Hyperscattering features of burned tissue on top of the ablated area and hyposcattering features of typical BE glands at the edge of the ablated area can be observed.
Supplementary Video 2: Complete cross-sectional fly-through of the volumetric OCT images obtained immediately after the CSA treatment. The spongiform appearance due to the edematous tissue caused by freezing can be observed in the data set.

## Figures and Tables

**Table 1:** Summary of the comparison between RFA and CSA.

Treatment method	RFA	CSA
Number of patients	10	3
Characteristics	Burned tissues with hyperscattering desquamation and debris	Edema-like spongiform appearance and minimal hyperscattering desquamation caused by freezing
Maximum depth of architectural changes (Mean ± SD)	(i) 263.33 ± 31.4 *μ*m for the first set of ablations (ii) 237 ± 34.9 *μ*m for the second set of ablations	(i) 643 ± 30 *μ*m for the whole treatment
